# Policy Modeling Consistency index-based study on policy synergy for sustainable artificial intelligence in China’s digital cultural industries

**DOI:** 10.1371/journal.pone.0345004

**Published:** 2026-03-16

**Authors:** Chen Qu, Xinyang Zhao

**Affiliations:** 1 Shanghai Research Institute for Intelligent Autonomous Systems, Tongji University, Shanghai, China; 2 Institute of Global Urban Civilisation, Southern University of Science and Technology; Shanghai Jiao Tong University, CHINA

## Abstract

This paper applies the Policy Modeling Consistency (PMC) index model to quantitatively evaluate 32 Chinese policies (2016–2025) promoting artificial intelligence (AI) in digital cultural industries (DCIs). Focusing on policy synergy for sustainable AI, the analysis assesses ten primary variables—including policy objectives, instruments, sustainability integration, and application levels—across national and local initiatives. Results reveal an overall ‘Excellent’ average PMC score, indicating robust policy design in economic or technological domains. However, some low-grade policies prioritize short-term regulation over sustainable governance. The study recommends embedding ‘human-centricity’ and ‘ethical risk assessment’ in sustainability indicators, alongside cultural diversity safeguards (e.g., algorithmic support for intangible heritage). These optimizations can reframe AI as a socio-cultural enabler—not merely a technical tool—advancing equitable, innovative, and ecologically resilient DCIs aligned with Sustainable Development Goals (SDGs).

## Introduction

Artificial intelligence (AI) is rapidly reshaping the global socio-economic landscape. Beyond enhancing efficiency through algorithms and computing power, AI is crucially a ‘socio-cultural technology’ [1], redefining how human knowledge and innovation are shared, transmitted and recreated. Within the framework of the Sustainable Development Goals (SDGs), the application of AI should transcend mere technical utility and focus on creating social value that promotes equity, innovation, and cultural inclusivity [2]. This process highlights the critical role of policy guidance, particularly in the integration of digital technology and creative industries. The deep interface of AI with cultural heritage, artistic creation, and media dissemination offers new pathways for the preservation, transmission, and recreation of collective human knowledge [3,4].

This paper evaluates and optimizes policies which highlights the role of sustainable AI within the context of China’s digital cultural industries (数字文化产业). According to China’s official document, the DCIs focus on cultural and creative content which rely on digital technology for creation, production, dissemination and services [5]. China is experiencing its digital transformation of cultural industries [6]. Particularly, China has recently implemented a series of innovative policies in the sector of DCIs to embed AI technology deeply within the cultural production and dissemination chain [7]. This includes using virtual reality (VR) to revitalize historical artifacts, employing generative AI to democratize content creation, and leveraging large models for precise international distribution of cultural products [8].

However, AI-driven cultural innovation also faces significant challenges, such as data privacy risks, algorithmic biases leading to information bubbles, and copyright disputes over AI-generated content [9]. These issues require systematic policy responses. In this context, this study utilizes the Policy Modeling Consistency (PMC) [10] index model to quantitatively evaluate China’s DCIs policies, aiming to reveal strengths and weaknesses in policy design and explore how to optimize policies to foster the synergistic development of AI technology and the cultural industries, ultimately achieving multidimensional sustainable development goals.

Beyond its empirical evaluation, this study offers a conceptual contribution to emerging debates on sustainable AI and socio-cultural technologies [1,11]. By applying the PMC model to policies governing AI in digital cultural industries, we demonstrate that AI in China is framed not only as a technological instrument but as a cultural and sustainability infrastructure that shapes how knowledge, values, and social practices are reproduced. This perspective extends existing discussions of sustainable AI, which often emphasize environmental efficiency or ethical risk, by foregrounding the cultural dimensions of AI governance and highlighting how policy design mediates the relationship between technological innovation, cultural vitality, and long-term socio-ecological goals. The PMC analysis reveals structural imbalances in policy orientation, showing that economic and technological objectives consistently outweigh cultural and sustainability considerations. This finding enables us to propose a normative framework that integrates human-centric governance, cultural diversity protection, and ethical risk assessment into the design of sustainable AI policies. Through this theoretical lens, the paper advances the understanding of AI as a socio-cultural technology and illustrates how policy systems can more effectively align AI innovation with cultural sustainability and the SDGs.

## Literature review

### Sustainable artificial intelligence: Concepts, opportunities, and risks

AI has been widely recognized as the driver of sustainable development, with scholars arguing that AI can enhance efficiency, reduce environmental impact, and address complex global challenges [2,12]. From applications in energy management to healthcare systems, AI contributes to multiple Sustainable Development Goals (SDGs), offering opportunities to optimize resource use, improve decision-making, and bridge global development gaps [13,14]. The World Economic Forum has mapped hundreds of technology applications to SDGs, underscoring AI’s relevance across environmental, economic, and social dimensions [15]. However, AI’s rapid integration into society also generates new risks: resource-intensive computation, algorithmic opacity, social bias, and inequalities stemming from uneven technological access [16]. These challenges illustrate that AI’s sustainability contributions cannot be understood as purely technical achievements but account for broader socio-economic impacts.

To address these tensions, some researchers have advanced the framework of Sustainable Artificial Intelligence (SAI), which considers AI both as a tool for achieving SDGs and as a system requiring governance to ensure its own sustainable use [11,17]. SAI research emphasizes human-centricity, accountability, environmental responsibility, and ethical design, highlighting the need for multi-level governance mechanisms to prevent undesirable societal outcomes [18]. Farrell, Gopnik (1) argue that AI should be analyzed not merely as an intelligent agent but as a socio-cultural technology, embedding human knowledge, norms, and institutional logics. This perspective suggests that successful SAI implementation depends on organizational culture, regulatory structures, and societal expectations—not only on technological capability [19].

### Cultural sustainability and AI in the digital age

The integration of digital technologies into cultural sectors has transformed the production, dissemination, and preservation of cultural knowledge. In China, the concept of cultural industries is derived from creative industries, and sometimes the two are used interchangeably [20]. As a later coined term, digital cultural industries (DCIs) emerged as a response to the growing role of digital tools in cultural innovation, heritage protection, and creative content production [21]. Similar global initiatives—including the UK’s CreaTech [22], Hong Kong’s Arts+Tech strategy [23], and UNCTAD’s Creative Industries 4.0 [24]—likewise position digital technologies as engines of cultural sustainability. These frameworks collectively suggest that AI and related technologies (e.g., VR, AR, generative models) play a pivotal role in reimagining cultural participation, enabling immersive heritage experiences, and expanding creative capacities [3,4].

However, the cultural implications of AI also raise concerns. Biases embedded in datasets risk reinforcing dominant cultural narratives, potentially marginalizing dialects, minority cultures, or intangible heritage [7,25]. Algorithmic personalization may narrow cultural diversity by prioritizing commercially successful or mainstream content. Moreover, intellectual property and authenticity debates surrounding AI-generated cultural content complicate long-term sustainability [9]. Scholars have therefore called for culturally sensitive AI governance frameworks that recognize both the creative possibilities and socio-cultural risks of digital transformation.

In China, these tensions are particularly pronounced due to strong state interest in leveraging AI for soft power, digital innovation, and industrial upgrading [26,27]. National strategies increasingly emphasize integrating AI across the cultural value chain—from heritage digitization to intelligent content distribution to VR-enhanced cultural industries [28]. At the same time, cultural institutions are experimenting with AI-generated cultural products, raising questions about cultural authenticity, community participation, and long-term sustainability [29].

The discussion on cultural sustainability in the era of AI highlights that AI’s value in cultural industries cannot be evaluated solely through economic or technological metrics. Instead, policies must be assessed for their support of cultural diversity, heritage preservation, equitable participation, and intergenerational transmission of cultural knowledge. These considerations directly inform the selection and interpretation of the policy evaluation related to sustainability and policy functions.

### Policy evaluation and the PMC framework

This section concerns methods for evaluating public policies, particularly in complex and multi-dimensional domains such as AI governance and cultural industries. Traditional approaches—including logic models, regulatory impact assessments, and qualitative content analysis—offer valuable insights but are often limited in their ability to capture multidimensional policy interactions [30,31]. The Policy Modeling Consistency (PMC) index, developed by Ruiz Estrada (10), addresses this gap by providing a systematic method for quantifying internal coherence across multiple policy components. The PMC model has been applied in diverse areas, including renewable energy, biotechnology, education, talent management, and social welfare [32,33]. Its flexibility makes it particularly suitable for analyzing policy synergy and internal structure, enabling researchers to compare policies across regions, time periods, or thematic domains.

Within cultural industry research, the PMC model has recently been adapted to assess policy design and coherence in China [34]. This emerging methodological trend demonstrates its growing relevance to cultural policy studies. Importantly, PMC’s structure—built around evaluating objectives, instruments, sustainability dimensions, and functions—aligns well with the theoretical pillars outlined above. It provides a way to assess whether policies articulate sustainability principles (as emphasized in SAI), incorporate cultural sustainability considerations, and deploy coherent policy tools to achieve desired outcomes.

Thus, the PMC index constitutes provides both the methodological rationale and the analytical lens through which China’s AI-related DCIs policies are evaluated. By bridging the conceptual insights from SAI and cultural sustainability with a quantitative evaluation model, this study integrates theoretical rigor with empirical precision.

## Methods

This research focuses on the quantitative research of policies related to China’s DCIs, employing the PMC index model to analyze policies from 2017 to 2024. It aims to construct targeted models, evaluation standards, calculation methods, and grading assessments for sustainable AI policies within the DCIs, proposing quantitative evaluation and optimization methods. The PMC index model was first introduced by Mario Arturo Ruiz Estrada [10]. This model helps researchers and decision-makers determine the consistency level of policies and assess the strengths and weaknesses of the policies to be evaluated within the policy index model. The PMC has been widely applied in sustainable research fields such as pharmaceutical policies, renewable energy environmental policies, educational policies, talent policies, and social policies [32,33]. Recently, it has also begun to be used in studies of cultural industries policies in China [34]; however, these studies have not addressed the impact of digital technology on the transformation of China’s cultural industries, and there is very little research specifically on sustainable AI in the context of China’s DCIs.

### Construction of the PMC index model

This paper defines the sample time for digital cultural industries policies as 2016–2025. The 2016 China Government Work Report [35] saw the central government propose the concept of ‘digital creative industries’, which was later amended in April 2017 to ‘digital cultural industries’. Furthermore, this paper outlines China’s DCIs policies, selecting those highlighting AI. This selection is based on the innovative, experimental, and demonstrative nature of AI policies during this period, which is conducive to constructing the PMC index model for quantitative evaluation and optimization. The primary sources for policy collection include the official website of the Central People’s Government, the National Bureau of Statistics, and local government portals, and supplemented by search engines like Peking University Law Database (北大法宝).

**Fig 1:**
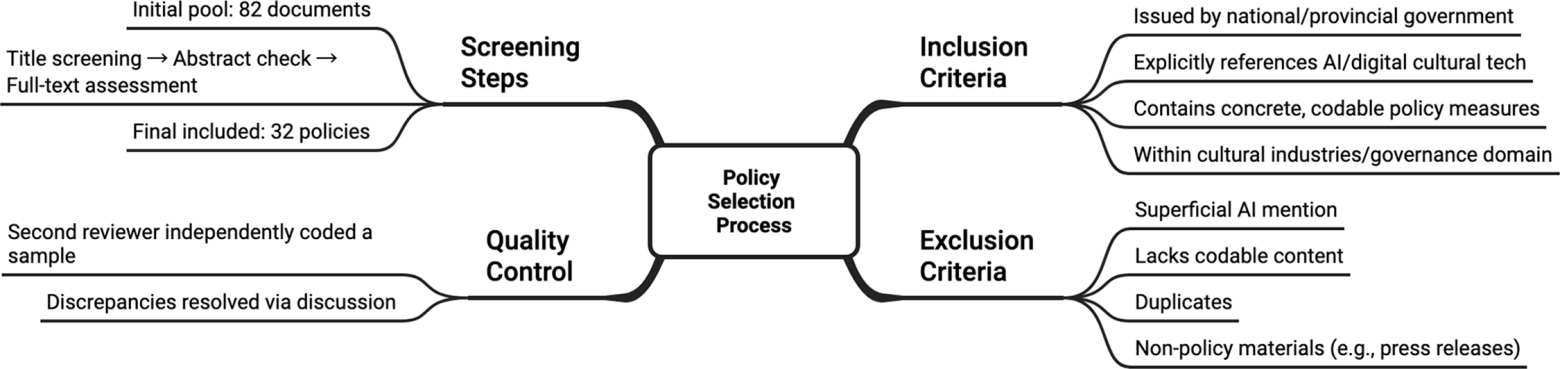
The process of policy selection. A flow diagram of the selection process of the document filtering procedure.

To ensure transparency and consistency in policy selection, explicit inclusion and exclusion criteria were applied [36,37]. A policy document was included if it (1) was issued by a national or provincial government authority, (2) explicitly referenced artificial intelligence or AI-enabled digital cultural technologies, (3) contained concrete policy measures suitable for PMC indicator coding, and (4) fell within the domain of cultural industries or cultural governance. Documents were excluded if they mentioned AI only superficially, lacked codable content, were duplicate announcements, or represented non-policy materials such as press releases or meeting summaries. Following these criteria, an initial pool of 82 documents underwent title-level screening, abstract-level relevance checks, and full-text assessment. 32 policies met all eligibility criteria and were included in the final analysis (see Table 1). To enhance coding reliability, a second reviewer independently assessed a sample of the policies using the predefined coding rubric, and discrepancies were resolved through discussion. A flow diagram of the selection process has been added to provide a clear overview of the document filtering procedure (Fig 1).

**Table 1:** 32 DCIs policy samples highlighting AI.

Code	Policy Title	Issuing Authority	Release Date
P1	Measures for the Identification of AI-Generated Synthetic Content	Cyberspace Administration of China, Ministry of Industry and Information Technology, Ministry of Public Security, National Radio and Television Administration	March 2025
P2	Development Plan for the New Generation of Artificial Intelligence	General Office of the State Council	July 2017
P3	Guiding Opinions on Promoting Deep Integration of Culture and Technology	General Office of the CPC Central Committee, General Office of the State Council	August 2019
P4	Opinions on Promoting the High-Quality Development of Digital Cultural Industries	Publicity Department of the CPC Central Committee, Ministry of Culture and Tourism	November 2020
P5	Ethical Norms for the New Generation of Artificial Intelligence	National Governance Committee for the New Generation of Artificial Intelligence	September 2021
P6	Science and Technology Development Plan for Radio, Television and Online Audiovisual During the 14th Five-Year Plan Period	National Radio and Television Administration	October 2021
P7	Development Plan for the Digital Economy During the 14th Five-Year Plan Period	National Development and Reform Commission	January 2022
P8	Guiding Opinions on Accelerating Scenario Innovation to Promote High-Level Application of AI for High-Quality Economic Development	Ministry of Science and Technology, Ministry of Education, Ministry of Industry and Information Technology, Ministry of Transport, Ministry of Agriculture and Rural Affairs, National Health Commission	July 2022
P9	Interim Measures for the Management of Generative AI Services	Cyberspace Administration of China	July 2023
P10	Three-Year Action Plan for Innovative Development of the Metaverse Industry (2023–2025)	Ministry of Industry and Information Technology	August 2023
P11	Policy Measures for Promoting High-Quality Development of the Digital Economy	General Office of the State Council	January 2024
P12	Shanghai Action Plan for Cultivating the ‘Metaverse’ New Track (2022–2025)	General Office of Shanghai Municipal People’s Government	June 2022
P13	Jiangsu Province Action Plan for Metaverse Industry Development (2024–2026)	General Office of Jiangsu Provincial People’s Government	October 2023
P14	Guiding Opinions of Zhejiang Provincial People’s Government on Accelerating the Development of AI Industry	General Office of Zhejiang Provincial People’s Government	January 2024
P15	Guiding Opinions of Shandong Provincial People’s Government on Accelerating the Implementation of ‘Ten Major Projects’ to Promote High-Quality Development of New Generation IT Industry	Shandong Provincial People’s Government	December 2023
P16	Digital Qingdao Development Plan	Qingdao Municipal People’s Government	May 2023
P17	Implementation Opinions of Beijing on Better Utilizing Data Elements to Further Accelerate Digital Economy Development	General Office of Beijing Municipal People’s Government	June 2023
P18	Action Plan for Innovative Development of the Metaverse in Beijing Sub-Center (2022–2024)	People’s Government of Tongzhou District, Beijing	August 2022
P19	Guangdong Action Plan for Cultivating Digital Creative Strategic Emerging Industrial Clusters (2021–2025)	General Office of Guangdong Provincial People’s Government	September 2020
P20	Smart Tourism Innovation Development Action Plan	Ministry of Culture and Tourism	May 2024
P21	Measures for Creating New Consumption Scenarios and Cultivating New Consumption Growth Points	Ministry of Commerce	June 2024
P22	Several Measures of Xi’an Municipality to Promote High-Quality Development of Digital Economy	Xi’an Municipal People’s Government	December 2023
P23	Implementation Plan for Promoting High-Quality Development of Digital Culture and Tourism in Fujian Province	General Office of Fujian Provincial People’s Government	April 2024
P24	Science and Technology Development Plan for Radio, Television and Online Audiovisual During the 14th Five-Year Plan Period	National Radio and Television Administration	August 2021
P25	Talent Development Plan for Radio, Television and Online Audiovisual During the 14th Five-Year Plan Period	National Radio and Television Administration	January 2023
P26	Notice on Implementing the Network Game Quality Publication Project	National Press and Publication Administration	November 2023
P27	National Strategic Emerging Industries Development Plan During the 13th Five-Year Plan Period	National Development and Reform Commission	December 2016
P28	Development Plan for Software and Information Technology Services Industry (2016–2020)	Ministry of Industry and Information Technology	January 2017
P29	Guiding Opinions on Promoting Innovative Development of Digital Cultural Industry	Ministry of Culture	April 2017
P30	Several Measures for Further Promoting the Development of Cultural and Creative Products by Cultural and Cultural Relics Institutions	Ministry of Culture and Tourism, National Cultural Heritage Administration	August 2021
P31	Chengdu 14th Five-Year Plan for Digital Cultural and Creative Industries Development	Chengdu Municipal People’s Government	January 2022
P32	Lv’an Digital Creative Industry Development Plan (2022–2035)	Lv’an Municipal People’s Government	August 2023

Following the variable settings proposed by Estrada, we selected ten primary variables: (X_1_) Policy Nature, (X_2_) Policy Timeframe, (X_3_) Policy Domain, (X_4_) Policy Objectives, (X_5_) Policy Instruments, (X_6_) Level of Application, (X_7_) Incentive Measures, (X_8_) Sustainable Development, (X_9_) Policy Functions, and (X_10_) Citation Recourse. Moreover, to ensure the maximal completeness and accuracy of statistical analysis, word segmentation was performed on 82 DCIs policies from 2016 to 2025 using the ‘Jieba’ library in Python, resulting in a list of the top 90 high-frequency terms including top 30 two-character Chinese words, to 30 three-character Chinese words and 30 four-character Chinese words (see Table 2). Based on high-frequency word statistics, secondary variables under X_4_ to X_9_ were established. The secondary variables for X_1_ to X_3_ and X_10_ are based on Estrada’s original model index and relevant PMC studies. Finally, this paper formed a PMC index model for evaluating digital cultural industries policies from 2016 to 2024, comprising 10 primary variables and 35 secondary variables (see Table 3).

Variables X_1_ to X_10_ represent nine primary dimensions for evaluating the role of AI in DCIs policies, forming a comprehensive assessment framework these policies.

**Table 2:** High-frequency word statistics.

Serial Number	Vocabulary	Frequency	Serial Number	Vocabulary	Frequency	Serial Number	Vocabulary	Frequency
1	Development	4728	31	Smart	899	61	Service System	144
2	Culture	3714	32	Digitalization	855	62	Talent Cultivation	142
3	Construction	3175	33	Internet	827	63	Standardization	138
4	Digital	2633	34	Socialism	650	64	Manufacturing	131
5	Data	2356	35	Cultural Industry	623	65	Public Data	126
6	Innovation	2303	36	Infrastructure	473	66	Networking	125
7	Service	2193	37	Informatization	449	67	Industrialization	122
8	Industry	2102	38	Digital Economy	420	68	Chinese Nation	121
9	Promotion	2054	39	Broadcasting and Television	397	69	Industrial Park	119
10	Enterprise	1943	40	Press and Publication	378	70	Economy and Society	116
11	Application	1835	41	Industrial Chain	346	71	Data Center	108
12	Strengthening	1790	42	Intelligentization	331	72	Innovation Capability	101
13	Technology	1703	43	High-Quality	294	73	Division of Responsibilities	101
14	Support	1443	44	Public Service	276	74	Strategic	100
15	Platform	1376	45	Modernization	252	75	Information Security	97
16	System	1358	46	Network Security	235	76	Interconnection	96
17	Enhancement	1335	47	Virtual Reality	225	77	Cultural Heritage	96
18	Acceleration	1302	48	Information Technology	200	78	Service Industry	95
19	Network	1246	49	Service Platform	190	79	Core Technology	95
20	Tourism	1178	50	Integration	188	80	Personalization	92
21	Nation	1173	51	Competitiveness	183	81	Robot	91
22	Integration	1160	52	Influence	181	82	Museum	87
23	Field	1155	53	Intellectual Property	173	83	Specialization	86
24	Intelligence	1073	54	Establishment and Improvement	172	84	Marketization	84
25	Product	1045	55	New Generation	163	85	High-Level	82
26	Improvement	1042	56	Technological Innovation	159	86	Supply Chain	77
27	Artificial Intelligence	997	57	National-Level	154	87	Industry-Academia-Research	73
28	Capability	943	58	Key Technology	151	88	Demonstration Zone	65
29	Encouragement	931	59	Data Security	147	89	New Energy	61
30	Resource	923	60	Full Utilization	147	90	Sustainable	60

**Table 3:** PMC evaluating variables.

Primary Variable	Secondary Variables	Source of Secondary Variables
X1 Policy Nature	X1:1 Normative X1:2 Guiding X1:3 Strategic/Pilot	Mario Arturo Ruiz Estrada (2011)
X2 Policy Timeframe	X2:1 Short-term X2:2 Medium-term X2:3 Long-term	Hu and Zhang (34)
X3 Policy Domain	X3:1 Culture X3:2 Technology X3:3 Economy X3:4 Society	Mario Arturo Ruiz Estrada (2011)
X4 Policy Objectives	X4:1 Technological Innovation X4:2 Consumption Expansion X4:3 Talent Development X4:4 Standardized Introduction	Based on high-frequency words in policy text mining
X5 Policy Instruments	X5:1 Financial Support X5:2 Regulatory Standards X5:3 Public Services X5:4 Market Cultivation	Based on high-frequency words in policy text mining
X6 Level of Application	X6:1 Central Level X6:2 Local Level X6:3 Industry Level	Based on high-frequency words in policy text mining
X7 Incentive Measures	X7:1 Talent Incentives X7:2 Financial Incentives X7:3 Technical Support	Based on high-frequency words in policy text mining
X8 Sustainable Development	X8:1 Green Development X8:2 Long-term Development X8:3 High-quality Development	Based on high-frequency words in policy text mining
X9 Policy Functions	X9:1 Industrial Integration X9:2 Technology Empowerment X9:3 Regional Cooperation X9:4 Collaborative Innovation	Based on high-frequency words in policy text mining
X10 Citation Recourse	--	Mario Arturo Ruiz Estrada (2011)

- X1 (Policy Nature) distinguishes between normative, guiding, strategic, and pilot policies, reflecting the fundamental positioning and function of each policy.
- X2 (Policy Timeframe) covers short-term, medium-term, and long-term policies, helping to assess implementation cycles and expected impacts.
- X3 (Policy Domain) identifies the main focus areas of the policy, such as culture, technology, economy, or society.
- X4 (Policy Objectives) clarify the intended goals, including technological innovation, expanding consumption, talent development, and regulation introduction, highlighting the policy’s key priorities.
- X5 (Policy Instruments) includes financial support, regulatory standards, public services, and market cultivation, providing practical instruments for policy implementation.
- X6 (Level of Application) refers to the level at which the policy is formulated and applied—national, local, or industry level—indicating the scope and extent of its influence.
- X7 (Incentive Measures) focuses on incentives related to talent, capital, and technology, which are crucial for enhancing the attractiveness and effectiveness of the policy.
- X8 (Sustainable Development) emphasizes green development, long-term development, and high-quality growth, reflecting the strategic foresight embedded in the policy.
- X9 (Policy Function) examines areas such as industrial integration, technological empowerment, regional cooperation, and collaborative innovation, showcasing the policy’s role in transforming and upgrading the cultural industry.
- X10 (Citation Recourse) evaluates whether relevant literature is cited within the policy to support its rationale and legitimacy.

Taken together, these variables form a systematic and structured framework for analyzing and categorizing DCIs policies.

### PMC index calculation and measuring method

The data in this paper is based on the content of policies, with corresponding values assigned to lower-level variables to derive the relevant data. This research uses Python scripts to assign values to each secondary variable and analyzes whether the high-frequency terms from the selected 32 DCIs policies align with the variable setting standards.

According to the principles established for the PMC index system, the following rules are defined: 1 for meeting the standard and 0 for not meeting the standard. For example, in the case of indicator X8 (Sustainable Development), each secondary indicator was designed based on high-frequency terms identified through policy text mining, while the specific scoring keywords were selected after reviewing all 32 policy documents and taking into account Chinese linguistic semantics and policy discourse conventions. Under X8, the secondary indicator X8:1 “Green Development” was operationalized using keywords such as “green” (绿色), “low-carbon” (低碳), and “environmental protection” (环保). The secondary indicator X8:2 “Long-term Development” was coded based on terms including “sustainability” (可持续), “long-term mechanism” (长效机制), and “future-oriented” (未来). The secondary indicator X8:3 “High-quality Development” was identified through keywords such as “high-quality” (高质量), “modernization” (现代化), “institutional soundness” (健全), and “diversification” (多元化). When one or more of these predefined keywords appeared in a policy document in a context consistent with the corresponding secondary indicator, the indicator was assigned a value of 1; otherwise, it was coded as 0. This rule-based and programmatic approach ensured consistency and reduced subjective interpretation in the scoring process.

Following the methodology proposed by Ruiz Estrada, the calculation model involves four steps: First, secondary variables are positioned as multiple inputs and outputs, and empirical analysis is used to assign values to these variables. Second, all secondary variables are constrained to follow a distribution within the [0,1] interval, with the assignment methods detailed in formulas (1) and (2). Third, as shown in formula (3), each first-level variable is calculated as the average sum of its corresponding secondary variables. Finally, formula (4) is applied to compute the PMC index for each indicator.


X~N[0,1] (1)

X={XR:[0~1]} (2)

$${\text{X}}_{\text{t}}\left(\int_{\text{j}=\text{1}}^{\text{n}}\frac{{\text{X}}_{\text{tj}}}{\text{n}}\right)\text{ t}=\text{1},\text{2},\text{3},\text{4},\text{5},\text{6},\text{7},\text{8},\text{9},\text{10}$$ (3)

t=一级变量 j=二级变量


$$PMC=\left[\begin{array}{c} @c@X_{\text{1}}\left(\sum_{i=1}^{\text{4}}\frac{X_{1i}}{\text{4}}\right)+X_{\text{2}}\left(\sum_{j=1}^{\text{3}}\frac{X_{2j}}{\text{3}}\right)+X_{\text{3}}\left(\sum_{k=1}^{\text{4}}\frac{X_{3k}}{\text{4}}\right)+\\ X_{\text{4}}\left(\sum_{l=1}^{\text{4}}\frac{X_{4l}}{\text{4}}\right)+X_{\text{5}}\left(\sum_{m=1}^{\text{4}}\frac{X_{5m}}{\text{4}}\right)+X_{\text{6}}\left(\sum_{n=1}^{\text{3}}\frac{X_{6n}}{\text{3}}\right)+\\ X_{\text{7}}\left(\sum_{o=1}^{\text{3}}\frac{X_{7o}}{\text{3}}\right)+X_{\text{8}}\left(\sum_{p=1}^{\text{3}}\frac{X_{8p}}{\text{3}}\right)+X_{\text{9}}\left(\sum_{q=1}^{\text{4}}\frac{X_{9q}}{\text{4}}\right)+X_{10} \end{array}\right]$$ (4)

Based on the above Formulas (1)-(4), this study calculated the PMC index scores of cultural industry policies. These scores were then used to classify all evaluated policies into graded categories. Since the evaluation framework incorporates 10 first-level variables, the PMC index ranges from 0 to 10. The classification criteria follow Estrada’s grading standard for cultural policy PMC index scores (Table 4).

**Table 4:** Classification criteria for PMC index scores of DCIs policies.

PMC Index Score	8 ~ 10	6 ~ 7.99	4 ~ 5.99	0 ~ 3.99
Policy Grade	Nearly Perfect	Excellent	Acceptable	Poor
Grade Code	A	B	C	D

To scientifically and comprehensively evaluate the DCIs policies, this research employs the PMC surface construction method based on the mentioned evaluation criteria and index scores for comparative analysis. The PMC surface visualizes all results contained in the PMC matrix, intuitively revealing the strengths and weaknesses of the policies under review within a multidimensional coordinate space, thereby more vividly reflecting the overall policy performance. The PMC surface is constructed based on a 3 × 3 variable PMC matrix, which encompasses independent scores for nine key variables. According to the classification of DCIs policy grades, the closer the PMC surface approaches the upper value of 1, the better the PMC index score of the policy. When the scores of the 3 × 3 variable matrix tend to be balanced and approach 1, the PMC surface displays an ideal symmetrical shape.

This paper defines a total of ten first-level variables, among which variable X_10_ represents the policy document reference value. Since none of the sampled policies mention reference documents, this variable scores 0 across all policies, lacking differentiation. The X10 (Citation Recourse) variable was removed from the PMC matrix because all sampled policy documents scored zero on this indicator. This uniformity reflects not a deficiency in the policies themselves, but a structural characteristic of Chinese policy writing [38], which rarely employs explicit citations or reference systems commonly found in Western governance documents. The absence of variance means that X10 does not contribute analytically to distinguishing policy performance, and retaining such a variable would distort the overall PMC evaluation. Therefore, variable X_10_ is excluded, and the PMC matrix is constructed using the scores of the remaining nine first-level variables, as shown in Formula (5). This approach effectively eliminates the interference of the policy document reference value on the overall score, allowing for a more accurate reflection of the intrinsic quality and structural characteristics of the policies.


$$\begin{array}{c} {PMC}_{surface}=(\begin{array}{ccc} X_{1} & X_{2} & X_{3}\\ X_{4} & X_{5} & X_{6}\\ X_{7} & X_{8} & X_{9} \end{array}) \end{array}$$ (5)

## Results: policy text rating and PMC surface drawing

### Policy text rating

Based on Formulas (1), (2), (3), and (4), this research calculates the PMC index scores for DCIs policies. Additionally, a P_avg_ value (average PMC score across 32 policies) was included. Following the grading criteria in Table 5, policies enacted between 2016 and 2020 were classified into respective grades.

**Table 5:** PMC Index and Grades of DCIs Policies.

Code	X1	X2	X3	X4	X5	X6	X7	X8	X9	X10	PMC Index	Rank	Grade
P01	0.25	0.00	0.75	0.50	0.25	0.33	0.33	0.00	0.25	0.00	2.67	33	D
P02	1.00	1.00	1.00	1.00	1.00	1.00	1.00	1.00	0.75	0.00	8.75	1	B
P03	1.00	0.00	1.00	1.00	0.75	1.00	1.00	0.67	0.75	0.00	7.17	23	B
P04	1.00	0.00	1.00	1.00	0.50	1.00	1.00	0.67	0.75	0.00	6.92	27	B
P05	1.00	0.00	0.75	0.75	0.25	0.67	0.33	0.33	0.50	0.00	4.58	31	A
P06	1.00	0.67	1.00	1.00	1.00	1.00	1.00	0.67	1.00	0.00	8.33	3	B
P07	1.00	0.33	1.00	1.00	1.00	1.00	1.00	1.00	1.00	0.00	8.33	6	B
P08	1.00	0.33	1.00	1.00	1.00	1.00	1.00	1.00	0.75	0.00	8.08	7	A
P09	0.75	0.00	1.00	0.75	0.25	0.33	0.33	0.33	0.50	0.00	4.25	32	A
P10	1.00	0.33	1.00	1.00	0.75	0.67	1.00	1.00	1.00	0.00	7.75	14	B
P11	0.75	0.33	1.00	1.00	1.00	1.00	1.00	0.67	1.00	0.00	7.75	15	B
P12	1.00	0.00	1.00	1.00	0.75	0.67	1.00	1.00	1.00	0.00	7.42	20	A
P13	1.00	0.33	1.00	1.00	0.75	1.00	1.00	1.00	1.00	0.00	8.08	8	A
P14	1.00	0.00	1.00	0.75	0.75	1.00	1.00	1.00	1.00	0.00	7.50	18	B
P15	1.00	0.00	1.00	1.00	0.75	0.67	1.00	0.67	1.00	0.00	7.08	24	A
P16	1.00	0.67	1.00	1.00	1.00	0.67	1.00	1.00	1.00	0.00	8.33	4	B
P17	1.00	0.67	1.00	1.00	1.00	1.00	1.00	0.33	1.00	0.00	8.00	11	A
P18	1.00	0.00	1.00	1.00	1.00	0.67	1.00	0.67	1.00	0.00	7.33	21	A
P19	1.00	0.00	1.00	1.00	0.75	0.67	1.00	0.33	1.00	0.00	6.75	29	C
P20	1.00	0.33	1.00	1.00	0.75	1.00	1.00	1.00	1.00	0.00	8.08	9	B
P21	0.75	0.00	1.00	1.00	1.00	1.00	1.00	1.00	1.00	0.00	7.75	16	A
P22	1.00	0.33	1.00	1.00	1.00	0.67	1.00	1.00	1.00	0.00	8.00	12	B
P23	1.00	0.00	1.00	1.00	1.00	1.00	0.67	0.33	1.00	0.00	7.00	26	B
P24	1.00	0.67	1.00	1.00	1.00	1.00	1.00	0.67	1.00	0.00	8.33	5	B
P25	1.00	0.33	1.00	1.00	1.00	1.00	0.67	1.00	1.00	0.00	8.00	13	B
P26	1.00	0.00	1.00	0.75	0.25	1.00	0.33	0.33	0.50	0.00	5.17	30	A
P27	1.00	0.33	1.00	1.00	0.50	1.00	1.00	1.00	0.75	0.00	7.58	17	B
P28	1.00	0.33	1.00	1.00	1.00	1.00	1.00	1.00	0.75	0.00	8.08	10	C
P29	1.00	0.33	1.00	1.00	0.75	1.00	1.00	0.67	0.75	0.00	7.50	19	A
P30	0.75	0.67	1.00	1.00	0.75	1.00	0.67	0.33	0.75	0.00	6.92	28	A
P31	1.00	0.33	1.00	1.00	0.75	0.67	0.67	0.67	1.00	0.00	7.08	25	A
P32	1.00	0.67	1.00	1.00	1.00	1.00	1.00	1.00	1.00	0.00	8.67	2	C
Pavg	0.97	0.29	1.00	0.98	0.84	0.88	0.91	0.77	0.93	0.00	7.57	22	B

**Fig 2:**
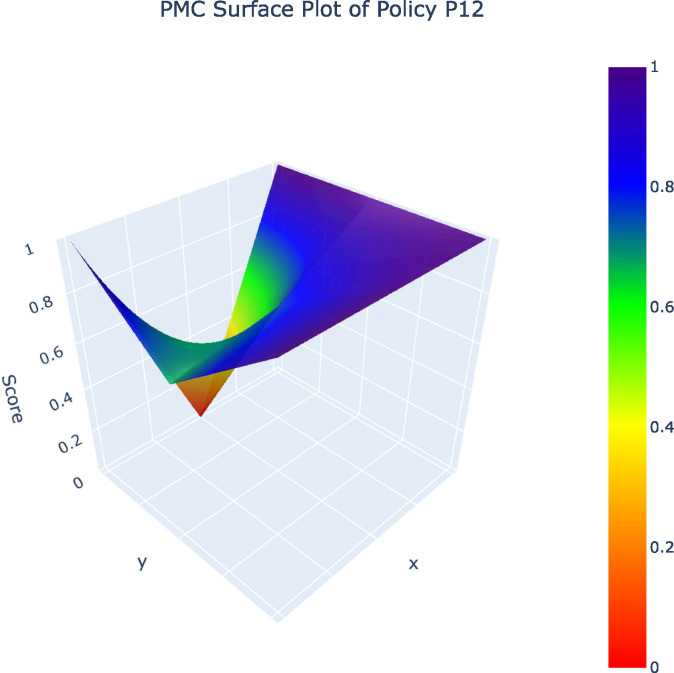
PMC Surface Plot of P12.PMC Surface Plot of Policy12.

**Fig 3:**
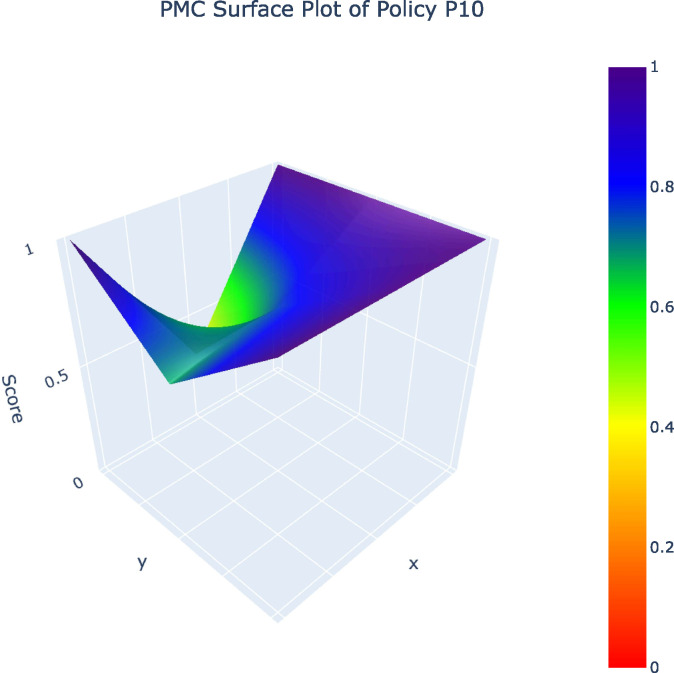
PMC Surface Plot of P10.PMC Surface Plot of Policy10.

**Fig 4:**
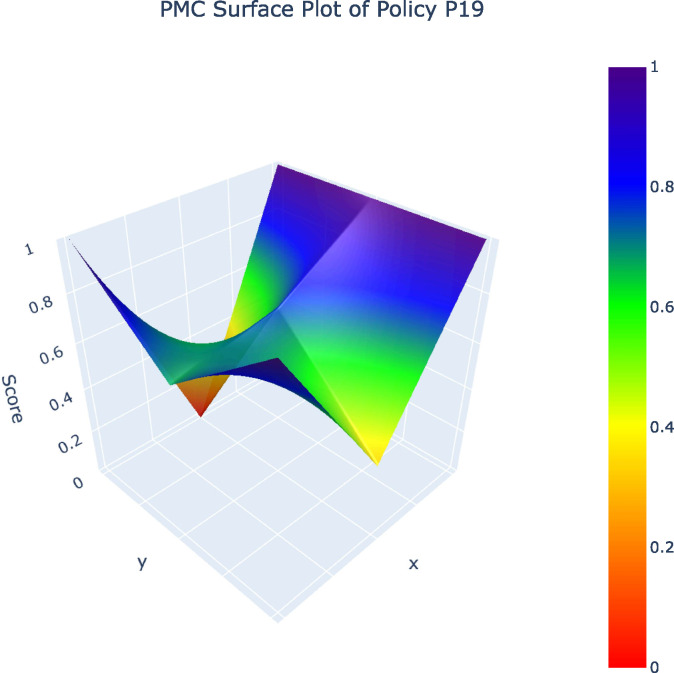
PMC Surface Plot of P19.PMC Surface Plot of Policy19.

**Fig 5:**
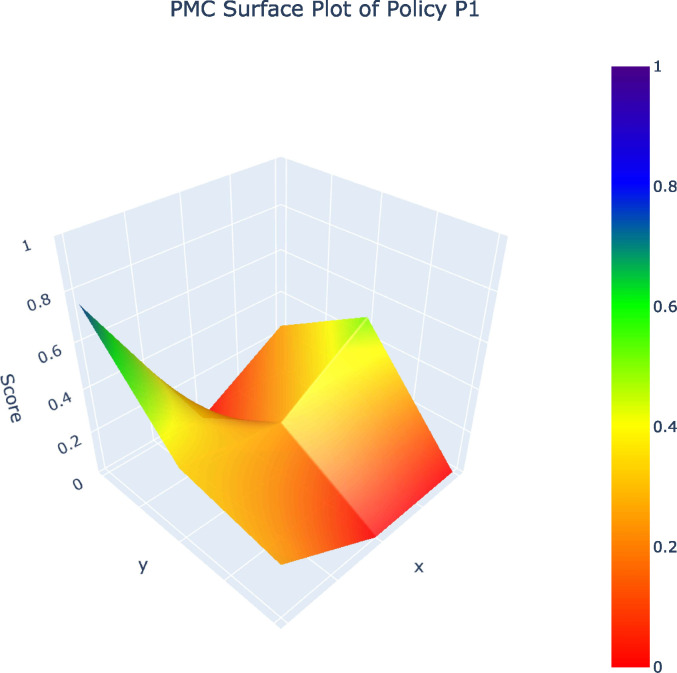
PMC Surface Plot of P1.PMC Surface Plot of Policy1.

**Fig 6:**
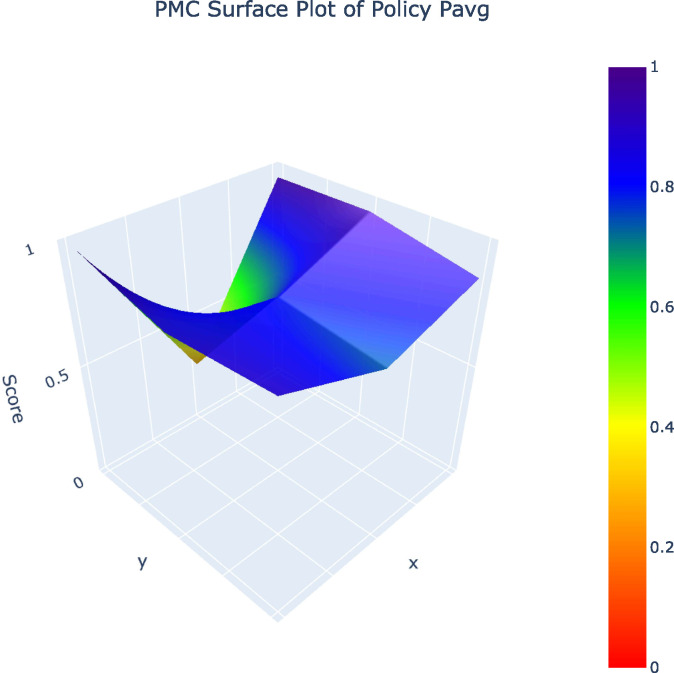
PMC Surface Plot of Pavg.The Surface Plot of the average PMC scores of the 32 polities.

From the 32 policy samples, four representative policies (P12, P10, P19, P1) – selected as one per grade (A, B, C, D) – along with the P_avg_ value were analyzed in PMC surface plots. The five PMC surface plots (Figs 2–6) were generated to comparatively examine variable-dimensional performance across policies.

To explore whether systematic differences exist in policy quality, additional subgroup analyses were conducted across issuing authority, region, and policy release period. Specifically, average PMC index scores were compared between policies issued by central and local governments, between earlier (2016–2022) and more recent (2023–2025) policy cohorts, and across major regional groupings (see Table 6). The regional groupings follow the conventional macro-regional classification in China by categorizing coastal regions with a high degree of openness and more mature economic and industrial foundations as eastern regions (e.g., Shanghai, Jiangsu, Zhejiang, and Guangdong), while classifying predominantly inland regions at a relatively earlier stage of development and primarily responsible for industrial transfer and regional coordination as central and western regions (e.g., Xi’an, Chengdu, and Lv’an).

**Table 6:** Comparison of average PMC Index scores across issuing authorities, time periods, and regions.

*Issuing Authority*	*PMC Index_avg*	*Issuing Date*	*PMC Index_avg*	*Issuing Region*	*PMC Index_avg*
*Central Government*	7.0995	*Earlier Period(2016–2022)*	6.9262	*Eastern Region*	7.4989
*Local Government*	7.6033	*Later Period(2023–2025)*	7.6506	*Western-middle Region*	7.9167

Overall, the results suggest that differences in average PMC scores across these dimensions are moderate rather than pronounced. Local government policies exhibit slightly higher average PMC scores than central government policies, and policies released in the later period show a modest improvement compared with earlier ones. Regional comparisons likewise reveal relatively close score distributions between eastern and central–western regions. Taken together, these patterns indicate that variations in policy quality are not driven by a single structural attribute, but instead reflect a broadly convergent policy design logic across issuing levels, regions, and time periods.

## Discussion: quantitative analysis of policy texts related to artificial intelligence in the digital cultural industry

The PMC-based evaluation of 32 policies governing AI in China’s DCIs reveals important structural patterns that carry significant policy implications. According to the analyses across issuing authority, region, and policy release period, the PMC results reveal not variation in policy quality, but a structural tendency toward techno-economic prioritization. The challenge is not uneven policy quality, but a shared structural blind spot.

Although the overall policy landscape demonstrates some strength, particularly in its emphasis on technological innovation, industrial development, and economic growth, the analysis also identifies persistent weaknesses in sustainability-related indicators. These gaps suggest that existing policies often conceptualize AI primarily as an engine of economic and technical modernization, rather than as a socio-cultural technology requiring long-term, human-centered governance [1,18]. As the results show, the sustainable development dimension (X8) scores consistently lower than other variables, indicating that environmental considerations, ethical safeguards, and cultural sustainability are not yet systematically embedded across policy instruments. This imbalance highlights a need to reorient AI governance frameworks so that they align more closely with the principles of SAI articulated in current scholarship [16,17].

The comparison of representative policies across A, B, C, and D grades illustrates these challenges more concretely. High-performing policies such as Shanghai’s Metaverse Action Plan (P12) embed sustainability by integrating green data centers, long-term development mechanisms, and coordinated fiscal tools, demonstrating that coherent policy design is achievable when sustainability is understood as foundational rather than supplementary. By contrast, lower-ranked policies, especially those falling into the C and D categories, relay heavily on regulatory controls or short-term incentives without establishing mechanisms for ethical concerns, cultural protection, or intergenerational equity. Such policies treat AI as an isolated technological intervention rather than as part of a broader socio-cultural system, an approach that risks undermining cultural diversity, exacerbating algorithmic bias, and narrowing the social benefits of AI [9,25].

These results point to several actionable suggestions for policymakers. Sustainability indicators should be expanded and standardized across national and local policy instruments. Current frameworks pay limited attention to cultural sustainability, despite clear evidence that AI reshapes aesthetics, creative practices, and heritage systems [7,39]. Policies could explicitly incorporate cultural diversity metrics, such as algorithmic support for dialect processing, minority cultural representation in datasets, or incentives for AI-driven intangible heritage revitalization. Embedding such indicators would ensure that DCIs grow in a manner that supports diversity and inclusivity.

Furthermore, the findings indicate that policy instruments need to be rebalanced to support long-term socio-cultural outcomes. While financial incentives and industrial development measures are widely deployed, they should be complemented with public service tools, community participation mechanisms, and transparent oversight structures. These instruments would enable policymakers to anticipate unintended consequences, strengthen accountability, and facilitate collaborative governance across cultural institutions and technology firms. Such an approach echoes broader recommendations in international SAI scholarship, which emphasize the social, behavioural, and institutional determinants of sustainable AI deployment [12,19].

Finally, although the present study focuses on China, the implications extend to global debates on AI governance. Many countries face challenges similar to those identified here: rapid technological adoption outpacing ethical regulation, uneven integration of sustainability principles, and growing tensions around cultural representation in AI systems. The PMC-based framework developed in this study offers a transferable method for evaluating policy coherence across diverse governance contexts. By quantifying gaps in sustainability, cultural protection, and long-term planning, the model can support international policymakers in designing approaches that balance innovation with social responsibility. Aligning national AI policies with global standards, such as the United Nations’ SDGs [40] and human rights–based AI frameworks [11] would further promote policy convergence and collective learning across regions.

## Conclusion

This study demonstrates that China’s AI-related policies for digital cultural industries possess a solid foundation in supporting technological advancement and economic modernization, yet reveal significant gaps in sustainability integration. While the high PMC scores in objectives, instruments, and technological empowerment indicate strong institutional momentum, the persistently weak performance in sustainability dimensions points to an underdeveloped awareness of AI’s broader socio-cultural and ethical implications. The evaluation of representative policies confirms that the most effective governance frameworks are those that embed sustainability principles across multiple policy dimensions linking long-term planning, cultural diversity, ethical oversight, and cross-sector collaboration. Such integration transforms AI from a productivity tool into a genuinely socio-cultural technology capable of supporting the multidimensional aims of the SDGs.

The insights generated by this study carry broader relevance beyond China. Many countries are grappling with similar tensions between rapid digital transformation and the need for responsible, inclusive governance. The PMC index, by offering a structured means of diagnosing inconsistencies and identifying areas for improvement, provides a valuable evaluative framework for policymakers worldwide. Its multidimensional approach makes it particularly well suited for sectors, such as cultural industries where technological, social, ethical, and cultural factors intersect. By adopting evaluation tools that highlight not only technological readiness but also cultural and sustainability considerations, policymakers can better shape AI development toward equitable and culturally grounded futures.

In moving forward, China’s DCIs policies, and international AI governance more broadly, would benefit from incorporating explicit sustainability metrics, algorithmic accountability mechanisms, and cultural diversity safeguards. Such measures align with emerging visions of AI as a socio-cultural technology [1] and support global efforts to create human-centered, ethically grounded digital ecosystems. Strengthening these dimensions will not only enhance policy coherence but also ensure that AI contributes meaningfully to cultural vitality, social well-being, and long-term sustainable development.

At the same time, this study has limitations. The PMC model evaluates policy coherence but cannot fully capture implementation outcomes, local enforcement dynamics, or stakeholder perspectives. The coding of sustainability and cultural indicators, though grounded in text-mined evidence, is necessarily interpretive, and future research would benefit from triangulating PMC results with interviews, case studies, or longitudinal impact assessments.

Overall, this research underscores the need for AI policies that integrate sustainability, cultural inclusivity, and ethical governance as core principles rather than peripheral considerations. Future studies should build on the PMC approach to examine multi-level policy interactions, track changes across time, and contribute to the development of globally informed frameworks for sustainable and culturally responsible AI governance.
